# 高效液相色谱-蒸发光散射法同时测定化妆品和牙膏中6种多羟基化合物

**DOI:** 10.3724/SP.J.1123.2024.02019

**Published:** 2025-03-08

**Authors:** Shu’e ZHAO, Lu YUAN, Dandan LIAO, Xiang LUO, Gengpeng XIAO

**Affiliations:** 江西省检验检测认证总院检测认证技术发展研究院, 江西 南昌 330029; Development Research Institute of Testing and Certification Technology, Jiangxi General Institute of Testing and Certification, Nanchang 330029, China

**Keywords:** 高效液相色谱, 蒸发光散射检测, 多羟基化合物, 化妆品, 牙膏, high performance liquid chromatography (HPLC), evaporative light-scattering detection (ELSD), polyhydroxy compounds, cosmetics, toothpaste

## Abstract

建立了同时测定化妆品和牙膏中玻色因、木糖醇、山梨醇、甘露醇、蔗糖、肌醇等6种多羟基化合物的方法。水可分散样品和水包油样品用水提取,提取液再经乙酸乙酯和正己烷洗涤;油包水样品先用乙酸乙酯预分散,再用水提取,最后提取液经正己烷洗涤。提取液经Ultimate XB-NH_2_柱(250 mm×4.6 mm, 5 μm)分离,乙腈-水溶液梯度洗脱,蒸发光散射检测器检测,外标法定量。分别对提取过程和色谱条件等进行了优化。在优化的实验条件下,6种多羟基化合物在0.2~5.0 g/L范围内线性关系良好,相关系数为0.991~0.996;方法的检出限(LOD, *S/N*=3)和定量限(LOQ, *S/N*=10)分别为0.10%和0.35%。针对水包油、油包水的化妆品和牙膏基质,进行了低、中、高3个水平的加标回收试验,目标物的平均回收率为84.7%~94.1%,相对标准偏差(RSD, *n*=6)为2.2%~6.9%。该方法具有经济简便、稳定可靠、重复性好等优势,适用于化妆品中多羟基化合物的检测。

## Abstract (en)

With the full implementation of the cosmetic formula ingredient registration system and the standardized management of labels and identification, the ingredients registered and declared on the label must be consistent with the actual ingredients contained in the product. Further, cosmetic manufacturers require accurate determination of the ingredients in cosmetics for formula analysis. Therefore, a method for the simultaneous determination of six polyhydroxyl compounds, Pro-Xylane, xylitol, sorbitol, mannitol, sucrose, and inositol, in cosmetics and toothpaste was established. According to this method, approximately 0.5 g of the sample was weighed into a 25 mL centrifuge tube. Water-dispersed and oil-in-water cosmetic samples were extracted using 10 mL water, followed by washing with 5 mL *n*-hexane and 5 mL ethyl acetate. The water-in-oil cosmetic samples were predispersed in 5 mL ethyl acetate, extracted using 10 mL water, and washed with 5 mL *n*-hexane. After filtration through a 0.22 μm membrane, the extracted solution was subjected to separation using an Ultimate XB-NH_2_ column (250 mm×4.6 mm, 5 μm), employing gradient elution with acetonitrile and distilled water as the mobile phases. This was followed by detection using evaporative light-scattering detector (ELSD) and quantification using the external standard method. Optimization experiments were conducted to select chromatographic columns, extraction processes, and chromatographic conditions. Specifically, the Ultimate XB-NH_2_ column and Ultimate XB-C_18_ column were compared in terms of separation efficiency. Based on the separation efficiency and analysis time, the XB-NH_2_ column was found to be more suitable as the separation column for this method. The effects of trichloromethane (TCM), ethyl acetate (EA), and *n*-hexane as predispersants and washing solutions were compared. Among them, the combination of ethyl acetate and *n*-hexane was found to be more suitable owing to its effective pre-dispersion capability and ability to remove both polar and nonpolar compounds from the extraction solution. The instrumental conditions were optimized using the gradient elution mode with acetonitrile and water as the mobile phase. The column temperature was set at 20 ℃, while the drift tube temperature and atomized gas pressure were set to 60 ℃ and 0.17 MPa, respectively. Under the optimized conditions, the method was validated in terms of linearity, sensitivity, recovery, and repeatability. The results showed that the six polyhydroxyl compounds exhibited excellent linearities within the range of 0.2-5.0 g/L, with correlation coefficients in the range of 0.991-0.996. Sensitivity is expressed as the limits of detection (LODs, *S/N*=3) and quantification (LOQs, *S/N*=10). The LODs and LOQs were 0.10% and 0.35%, respectively. Recovery tests were conducted at three levels (low, medium, and high) for oil-in-water cosmetics, water-in-oil cosmetics, and toothpaste matrices. Samples were extracted and measured six times in parallel. The average recoveries of the analytes were 84.7%-94.1%, with relative standard deviations (RSDs, *n*=6) of 2.2%-6.9%. Finally, the proposed method was employed to analyze six polyhydroxyl compounds in cosmetics and toothpaste that were randomly purchased from a local market. Sorbitol and xylitol were found to be the most commonly used ingredients in toothpaste, while Pro-Xylane was more common in whitening cosmetics. This method has the advantages of simplicity, stability, reliability, and good reproducibility and is suitable for the detection of polyhydroxy compounds in cosmetics. This method effectively addresses the current issue of inadequate detection of polyhydroxyl compounds in cosmetics.

多羟基化合物(polyhydroxy compounds, PHCs)是指分子中含有两个以上羟基的化合物。因富含羟基,具有良好的水溶性和吸湿性,在各类化妆品中均有广泛应用^[1,2]^。根据沸点不同,PHCs可以分为低沸点和高沸点两类。其中低沸点类主要有C3~C10的二元醇、甘油、乙基己基甘油等,具有结构简单、性质稳定、沸点较低等特点,可以直接汽化进行气相色谱测定^[3,4]^。高沸点类主要有聚醚多元醇、糖醇类、聚酯多元醇等。此类化合物结构复杂,性质迥异,且沸点高,极性大,使得测定方法也多样化。依据《已使用化妆品原料目录》和相关文献报道,目前化妆品和牙膏中使用的PHCs主要有玻色因、木糖醇、山梨醇、甘露醇、蔗糖、肌醇等,分别在配方中作为抗衰修复剂、保湿剂、甜味剂和功能活性物质^[2,5,6]^。2021年实施的新版《化妆品监督管理条例》要求全面实行化妆品配方备案注册制,同时还加强了对化妆品标签标识的规范化管理,要求备案的成分、标签上声明的成分与产品中实际含有的成分三者保持一致^[7]^。另一方面,随着原始委托制造商(OEM)、原始设计制造商(ODM)和原始品牌制造商(OBM)等全产业链加工模式在化妆品行业中的盛行,配方分析和产品仿制是快速研发新产品,抢占市场先机的重要手段,同时也是生产厂家提升研发实力,提高产品质量的最快路径。在此背景下,准确测定化妆品和牙膏中PHCs的含量很有意义。

目前,关于PHCs检测方法的研究多集中在药品、食品领域,已见报道的测定方法有高效液相色谱法(HPLC)^[8⇓-10]^、气相色谱-氢火焰离子化法(GC-FID)^[11⇓-13]^、离子色谱法-积分脉冲安培法(IC-IPAD)^[14⇓⇓-17]^、气相色谱-质谱法(GC-MS)^[18,19]^和高效液相色谱-串联质谱法(HPLC-MS/MS)^[20,21]^。其中GC-FID和GC-MS由于PHCs沸点较高,难以汽化,常常需要衍生为低沸点的化合物进行测定,整个过程复杂,操作繁琐。IC-IPAD容易使PHCs在电极表面发生氧化还原反应从而影响方法的准确性。HPLC-MS/MS灵敏度和特异性有明显优势,但是仪器价格较为昂贵,难以大范围推广。由于PHCs结构中缺乏发色团,没有特定的紫外吸收,需要使用独特的检测器。示差折光检测器(RI)^[22]^、蒸发光散射检测器(ELSD)^[23]^和电雾式检测器(CAD)^[24]^是目前常用的检测器。其中RI对温度敏感且不能用于梯度洗脱分析,在灵敏度和再现性方面有很大的局限性,不适于多种化合物的同时分离和检测。CAD灵敏度较高,重复性和稳定性好,是近年来推广应用的新型检测器,但整体普及率不高,且价格偏高。同CAD相比,ELSD同样具有基线稳定、灵敏度较高、重复性好等特点,是PHCs分离和定量分析的理想方法,可用于多种PHCs的同时测定。

经检索发现,目前文献报道的关于化妆品中PHCs的测定主要集中于玻色因、肌醇和山梨醇^[11,25,26]^,方法的检测项目过于单一,不够全面,未能进行同时测定;且未见有蔗糖、木糖醇和甘露醇测定的相关研究报道。基于此,参考文献[8,9,23],本文采用HPLC-ELSD建立了同时测定化妆品和牙膏中6种PHCs的方法。针对水包油(O/W)、油包水(W/O)体系化妆品和牙膏,优化了前处理过程和仪器方法条件。该方法具有操作简单、方法准确、回收率高、重复性好等优点,具有一定的应用前景。

## 1 实验部分

### 1.1 仪器与试剂

2695高效液相色谱分离单元-2424蒸发光散射检测器(美国Waters公司); Vortex 3型涡旋混合器(德国IKA公司); HC-2062高速离心机(科大创新公司); KQ-500DE超声波清洗仪(昆山市超声仪器有限公司); Mettler Toledo AL204电子分析天平(瑞士Mettler Toledo公司); Milli-Q超纯水器(美国Millipore公司)。

玻色因(含量≥98%)、木糖醇(含量≥99%)、山梨醇(含量≥98%)、甘露醇(含量≥98%)、蔗糖(含量≥99%)和肌醇(含量≥99%)均购于上海麦克林生化科技有限公司。正己烷、乙酸乙酯、乙腈(色谱纯)购于上海安谱实验科技股份有限公司,实验用水(超纯水)自制。

### 1.2 标准溶液的配制

称取PHCs各1 g(精确到0.0001 g),置于25 mL容量瓶中,用水溶解并定容,摇匀,即得40 g/L混合标准储备液。采用铝箔避光保存于4 ℃冰箱中,有效期半年。

分别移取不同体积的混合标准储备液用水逐级稀释成含6种PHCs质量浓度0.2、0.5、1.0、2.0、5.0 g/L的系列标准工作溶液。

### 1.3 仪器条件

色谱柱:Ultimate XB-NH_2_柱(250 mm×4.6 mm, 5 μm,上海月旭科技股份有限公司);柱温:20 ℃。流动相:(A)乙腈和(B)水。梯度洗脱程序:0~5 min, 80%A; 5~24 min, 80%A~70%A; 24~25 min, 70%A~80%A。流速1.0 mL/min,进样体积20 μL。ELSD参数:喷雾器模式为冷却模式,漂移管温度为60 ℃。载气为高纯氮气(99.999%),氮气压力为0.17 MPa,增益为100。

### 1.4 样品前处理

称取0.5 g(精确到0.001 g)样品于25 mL离心管中。

水可分散型及O/W型化妆品、牙膏:在样品中准确加入10 mL水,涡旋混合2 min后,然后超声提取20 min。提取液于5000 r/min离心10 min后,刮去上层油脂。再加入5 mL乙酸乙酯涡旋振荡洗涤,离心后弃去有机相,水相用5 mL正己烷涡旋振荡重复洗涤一次,离心,取水相,过0.22 μm滤膜,进样分析。

W/O型化妆品:在样品中先加入5 mL乙酸乙酯涡旋预分散,再准确加入10 mL水,然后涡旋提取10 min。提取液于5000 r/min离心10 min后,刮去上层油脂,弃去有机相。水相再加入5 mL正己烷洗涤,离心,取水相,过0.22 μm滤膜,进样分析。

## 2 结果与讨论

### 2.1 仪器条件的优化

目前用于PHCs检测的色谱柱主要有NH_2_柱和糖类分析专用柱^[10,23]^。有报道称糖类分析专用柱在高比例水相流动相的洗脱下易造成柱流失,使得分离度变差,基线不稳^[23]^。在条件优化的初期,我们比较了Ultimate XB-C_18_柱和Ultimate XB-NH_2_柱对PHCs分离的影响。PHCs在Ultimate XB-C_18_色谱柱上均无很好的保留,而使用Ultimate XB-NH_2_柱分离时则更有优势。可能是因为PHCs亲水性强,在Ultimate XB-NH_2_柱上表现出强弱不同的氢键结合力。因此,本实验选择Ultimate XB-NH_2_柱进行色谱分离。

由于PHCs易溶于水,难溶于乙腈,为防止分析物在进样后析出,乙腈和水的混合物常常用作流动相。实验考察了不同体积比(80∶20、 70∶30, v/v)的乙腈-水作为流动相对6种PHCs分离的影响。结果发现增加乙腈比例有利于目标物的分离,而增加水相比例可以缩短分析时间,同时基线噪声也会随之增大。因此本实验采用梯度洗脱,在5 min时开始逐渐降低有机相比例,有利于蔗糖和肌醇的洗脱,在25 min内基本实现6种PHCs的分离(图1)。实验还发现色谱柱温度对目标物的分离也有影响,温度高有利于分析物快速流出,但不利于山梨醇和甘露醇的分离。经考察,本方法设定色谱柱温度为20 ℃。实验还考察了ELSD漂移管温度和载气压力。根据流动相选择合适的漂移管温度,温度过高易引起流动相沸腾,噪声大,严重时导致部分组分汽化。温度过低,流动相蒸发不完全。载气压力在保证流动相蒸发完全的情况下尽量偏小设置,从而使得形成的溶质颗粒越大,其散射光强度也越强。通过逐渐改变漂移管温度或载气压力,观察基线噪声和响应信号强弱来优化ELSD的设置。实验发现,当漂移管温度为60 ℃、载气压力为0.17 MPa、喷雾器模式冷却时,噪声最小,响应信号最强。故确定此条件为最佳条件。

**图1:**
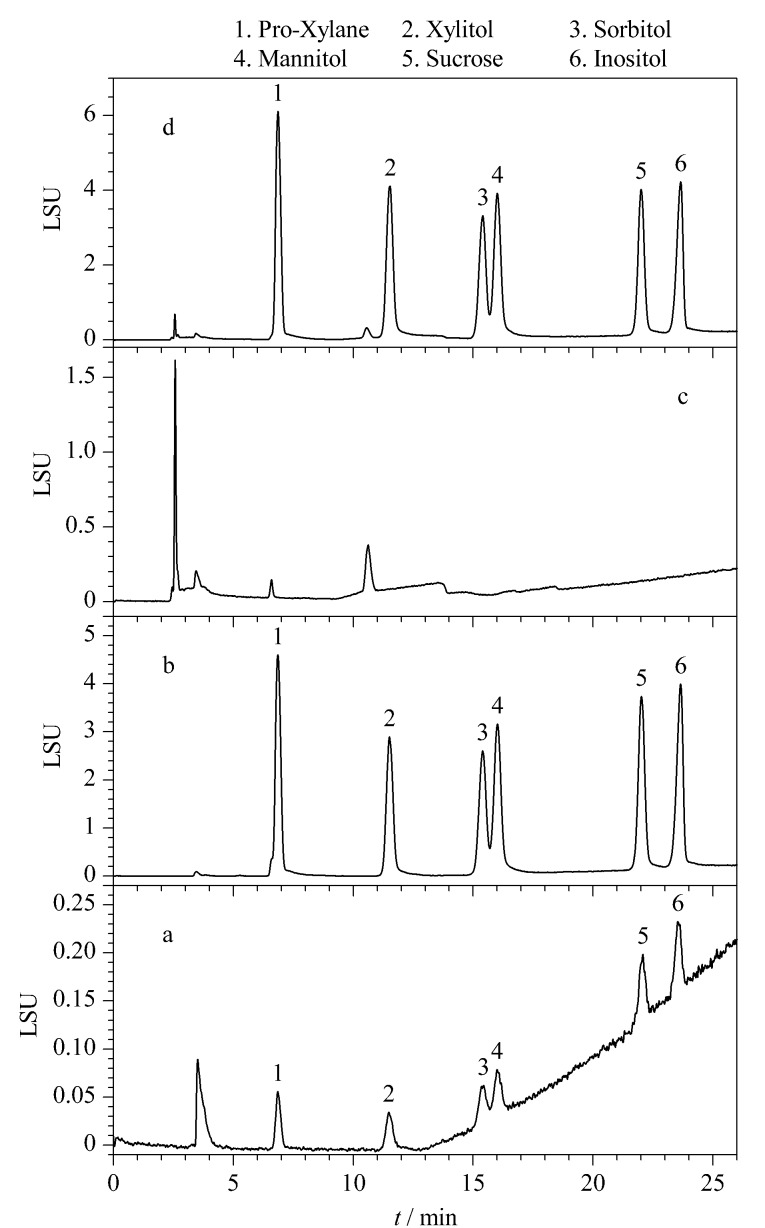
(a)0.05 g/L PHCs标准溶液、(b)0.2 g/L PHCs标准溶液、(c)空白样品和(d)加标样品的色谱图

### 2.2 前处理条件的优化

PHCs分子结构中含有多个羟基,由于羟基的氢键作用,使得其极易溶于水,不溶于有机溶剂。因此本文考虑用水作为提取溶剂,一方面可以有效地提取目标物,另一方面还可以祛除样品中大量的油脂成分和高分子化合物。经实验发现O/W体系化妆品和牙膏都有很好的提取效率。W/O体系化妆品由于样品无法充分分散,造成提取效率较差。因此需要添加亲油性溶剂进行破乳预分散,再用水进行萃取。对比了三氯甲烷、正己烷、乙酸乙酯对样品的预分散能力,实验发现3种溶剂都有很好的预分散能力。其中三氯甲烷毒性较大,渗透力强,易溶胀离心管;与正己烷相比,乙酸乙酯极性更大、渗透力也更强,使得样品更容易分散,且分散后黏度也更低。实验还发现由于化妆品中乳化剂的存在,使得提取液易产生乳化呈微乳状。为了避免进样时,提取液中的油脂和高分子化合物堵住进样针,需要对提取液进行洗涤。考虑到一般化妆品中既含有非极性化合物也含有极性化合物,因此选择非极性溶剂和极性溶剂的组合来进行洗涤。综合预分散实验结论,选择使用乙酸乙酯和正己烷进行预分散和洗涤。

### 2.3 方法验证

#### 2.3.1 线性范围和检出限

在优化的仪器条件下测定配制好的系列标准工作溶液,每个标准点测定3次,取峰面积平均值。以目标化合物平均峰面积的对数值为纵坐标(*y*),浓度的对数值为横坐标(*x*),采用最小二乘法进行曲线拟合,得到各目标物的线性方程和相关系数,相关参数结果列于表1中。结果表明,6种PHCs在0.2~5.0 g/L范围内均呈现良好的线性关系,其相关系数(*r*)为0.991~0996。灵敏度以方法检出限(LOD)和定量限(LOQ)来表示。采用在空白样品溶液进行加标试验获得,当加标溶液峰高响应至3倍和10倍空白溶液噪声时所对应的浓度为检出浓度和定量浓度,并由此计算得到PHCs的LOD和LOQ分别为0.10%和0.35%。

**表1:** 6种PHCs的保留时间、线性方程和相关系数(*r*)

Compound	Retention time/min	Linear equation^*^	r
Pro-Xylane	6.88	y=1.239x+0.772	0.995
Xylitol	11.53	y=1.275x+0.575	0.994
Sorbitol	15.42	y=1.224x+0.748	0.991
Mannitol	16.01	y=1.206x+0.895	0.993
Sucrose	22.01	y=1.166x+1.012	0.994
Inositol	23.65	y=1.128x+1.146	0.996

* Linear range: 0.2-5.0 g/L; *y*: logarithm of peak area; *x*: logarithm of concentration.

#### 2.3.2 回收率和精密度

在O/W、W/O体系的化妆品和牙膏的空白样品中均加入低、中、高3个水平的标准溶液,在优化的实验条件下对6种PHCs进行平行提取和测定6次,计算平均回收率和相对标准偏差(RSD),结果见表2。结果表明,PHCs在3类不同产品中的回收率为84.7%~94.1%, RSD为2.2%~6.9%。

**表2:** 不同配方体系化妆品中PHCs的加标回收率和精密度(*n*=6)

Compound	Spiked/mg	Recoveries/%				Intra-day precisions/%		
		O/W	Toothpaste	W/O		O/W	Toothpaste	W/O
Pro-Xylane	4.0	92.6	91.2	93.2		2.6	2.2	3.8
	20	93.1	92.5	91.5		2.4	2.8	3.1
	40	93.6	93.3	92.6		2.9	2.7	3.9
Xylitol	4.0	91.6	90.8	86.5		3.2	3.1	3.6
	20	93.5	92.1	90.8		2.9	3.5	3.4
	40	94.1	91.6	91.2		3.1	2.8	3.0
Sorbitol	4.0	90.7	86.7	84.7		4.6	4.7	5.6
	20	91.1	89.2	86.7		4.3	4.4	5.2
	40	90.7	91.7	89.2		4.0	4.6	5.5
Mannitol	4.0	89.9	87.4	90.1		6.4	6.2	6.9
	20	90.5	89.2	91.2		5.6	5.1	6.2
	40	91.0	90.8	90.4		5.2	5.4	6.0
Sucrose	4.0	89.3	90.5	88.5		4.2	4.6	4.3
	20	90.1	91.8	90.7		3.9	4.0	4.1
	40	91.2	92.6	91.8		3.7	3.7	4.2
Inositol	4.0	90.9	90.1	91.8		2.6	2.9	3.0
	20	92.3	91.2	91.2		2.8	3.0	3.2
	40	91.2	93.4	92.7		2.5	3.1	2.8

O/W: oil-in-water; W/O: water-in-oil.

### 2.4 实际样品检测

采用本方法对市场上随机购买的化妆品和牙膏样品共30份进行了检测,其中10份牙膏、10份O/W乳液、10份W/O膏霜,根据待测组分的含量决定是否对样品溶液进行稀释,使其进样浓度落在标准曲线范围内。结果显示共有4份样品检出含有PHCs,总体检出率为13.3%,检出量为0.51%~32.3%。3份牙膏检出含有山梨醇,含量分别为20.1%、32.3%和25.6%,其中含有25.6%山梨醇的牙膏样品还含有2.4%木糖醇; 1份O/W美白乳液检出含有玻色因,测定值为0.51%;其他样品均未检出PHCs。上述样品的检出情况与外包装标签标识一致。

## 3 结论

本工作建立了适用于不同配方体系化妆品和牙膏中6种PHCs同时测定的方法,样品采用水提取,经乙酸乙酯和正己烷预分散和洗涤后,HPLC-ELSD测定。该方法简便经济,回收率高,重复性好,已成功地应用于实际样品的检测,为目前化妆品中PHCs检测方法做了必要的补充。本方法的建立有助于化妆品生产公司进行配方分析。

## References

[1] Yan S X, Qu K H, Liu Y H, et al. China Surfactant Detergent & Cosmetics, 1995(6): 12

[2] Wang L L, Pan J F, Chen J Y. Food and Drug, 2022, 24(6): 600

[3] Tang Y P. Flavour Fragrance Cosmetics, 2020(1): 21

[4] Bao Z J, Wu W J, Quan X J, et al. China Surfactant Detergent & Cosmetics, 2010, 40(3): 229

[5] National Medical Products Administration. No. 62 Bulletin of the National Medical Products Administration (2021). (2021-05-01). https://www.nmpa.gov.cn/directory/web/nmpa/images/1686130272110055533.xlsxhttps://www.nmpa.gov.cn/directory/web/nmpa/images/1686130272110055533.xlsx

[6] Di L Q, Yu H, Chen J M, et al. Journal of Leshan Normal University, 2021, 36(12): 18

[7] The State Council of the People’s Republic of China. Regulations on the Supervision and Administration of Cosmetics (2020). (2020-06-16). https://www.gov.cn/zhengce/content/2020-06/29/content_5522593.htmhttps://www.gov.cn/zhengce/content/2020-06/29/content_5522593.htm

[8] Koh D-W, Park J-W, Lim J-H, et al. Food Chem, 2018, 240: 694 28946331 10.1016/j.foodchem.2017.07.142

[9] Zhang Y Q, Zhang W H, Hou J B, et al. J Chromatogr B, 2023, 1217: 123621 10.1016/j.jchromb.2023.123621 36746090

[10] Ding H L, Li C, Jin P, et al. Chinese Journal of Chromatography, 2013, 31(8): 804 24369618 10.3724/sp.j.1123.2013.01013

[11] Chen H R, Chen Y J, Han F, et al. Physical Testing and Chemical Analysis Part B: Chemical Analysis, 2014, 50(8): 1030

[12] Woollard D C, Macfadzean C, Indyk H E, et al. Int Dairy J, 2014, 37(2): 74

[13] Wu A Y, Song C Y, Xiang L X. Modern Food, 2022, 28(7): 182

[14] Zhang S F, Sheng H D, Jiang K, et al. Chinese Journal of Chromatography, 2016, 34(10): 946

[15] Guo Y J, Wang C M, Lin S X, et al. Chinese Journal of Pharmaceutical Analysis, 2022, 42(3): 525

[16] Xu W Y, Jiang Z B, Fan J, et al. Acta Pharmaceutica Sinica, 2023, 58(8): 2476

[17] Zheng J, Liu J Y, Shang J J, et al. Journal of Analytical Science, 2024, 40(1): 94

[18] Zhou H B, Xiong Z Y, Yu Y, et al. Chinese Journal of Chromatography, 2013, 31(8): 786 24369614 10.3724/sp.j.1123.2013.01005

[19] Xiang P, Tang Z. Journal of Chinese Mass Spectrometry Society, 2018, 39(3): 360

[20] Li Y, Liang J, Gao J N, et al. Carbohydr Polym, 2021, 272: 118478 34420737 10.1016/j.carbpol.2021.118478

[21] Li Y X, He Z Y, Zou P, et al. Microchem J, 2023, 193: 109136

[22] Filip M, Vlassa M, Coman V, et al. Food Chem, 2016, 199: 653 26776021 10.1016/j.foodchem.2015.12.060

[23] Hu B, Wang S, Xie F W, et al. Chinese Journal of Chromatography, 2012, 30(3): 298 22715697 10.3724/sp.j.1123.2011.11014

[24] Grembecka M, Lebiedzińska A, Szefer P. Microchem J, 2014, 117: 77

[25] Wu C Q, Nie L, Yang J Q. Flavour Fragrance Cosmetics, 2017(3): 26

[26] Yu H Y, Qiao Z Y, Li Q Y, et al. China Surfactant Detergent & Cosmetics, 2023, 53(6): 721

